# A Man with Cyanotic Digits

**DOI:** 10.5811/cpcem.2017.2.33371

**Published:** 2017-07-06

**Authors:** Tess Wiskel, Jonathan Bass, Jeffrey Feden

**Affiliations:** * Brown University Warren Alpert Medical School, Department of Emergency Medicine, Providence, Rhode Island; † Brown University Warren Alpert Medical School, Department of Plastic and Reconstructive Surgery, Providence, Rhode Island

## CASE PRESENTATION

**Image 1:**
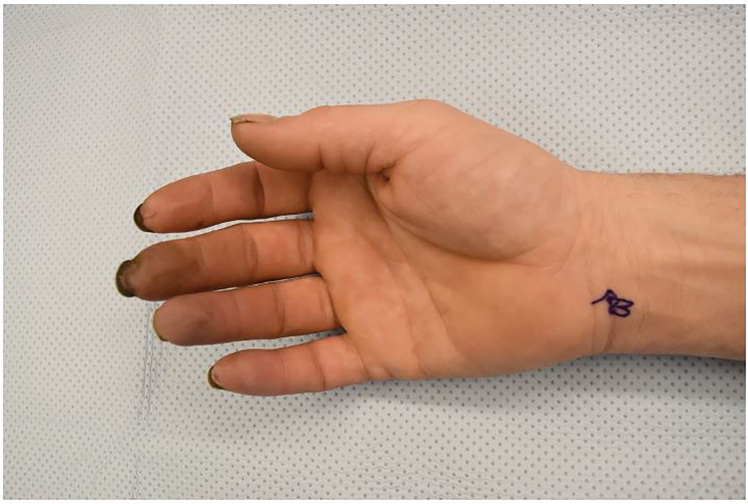
Initial appearance of hand with cyanotic digits 2–5.

A 37-year-old right-handed male auto mechanic with a 40 pack-year smoking history presented to the emergency department with progressive digital pain, cyanosis and paresthesias to his right hand over the prior month. Physical exam revealed cyanosis of the second through fifth digits with sparing of the thumb, absent digital artery Doppler signals, and a diminished deep palmar arch signal (Image 1).

Conventional angiography of the hand revealed an aneurysmal appearance of the palmar ulnar artery adjacent to the hook of the hamate, and abrupt truncations to the digital arteries consistent with hypothenar hammer syndrome (Image 2a). The patient returned two weeks later for surgical intervention with right ulnar artery reconstruction and ulnar nerve decompression (Image 2b). Following surgery, the cyanosis resolved and there was return of biphasic digital artery signals, normal capillary refill, motion, sensibility, and good wound healing.

## DISCUSSION

Hypothenar hammer syndrome is characterized by vascular insufficiency of the digits. It often results from repetitive blunt trauma to the hypothenar eminence causing ulnar artery damage, likely against the hook of the hamate, resulting in arterial thrombosis or aneurysm.^1^,^2^ This rare syndrome occurs most commonly in athletes and industrial workers.^3^ The differential diagnosis includes other causes of digital ischemia, such as Raynaud’s disease, Buerger’s disease, atherosclerotic and embolic disease, vasculitis, and thoracic outlet syndrome.^2^ The gold standard for diagnosis is arterial imaging, preferably angiography, which demonstrates the classic corkscrew or aneurysmal appearance of the ulnar artery. Additionally, point of care ultrasound has been used to aid in diagnosis of this syndrome.^4^ Management ranges from conservative medical management to endovascular thrombolysis or surgical grafting, as in this case.^2^

CPC-EM Capsule

What do we already know about this clinical entity?

Hypothenar hammer syndrome is characterized by vascular insufficiency to the digits from repetitive blunt trauma to the hypothenar eminence, which results in arterial aneurysm or thrombosis.

What is the major impact of the image(s)?

These images illustrate the physical exam findings of digital ischemia, the underlying vascular pathology demonstrated by angiography and the intra-operative gross anatomic dissection.

How might this improve emergency medicine practice?

Hypothenar hammer syndrome is a rare condition not often encountered in emergency medicine practice or literature, and these images intend to increase awareness in clinical practice.

## Figures and Tables

**Image 2a:**
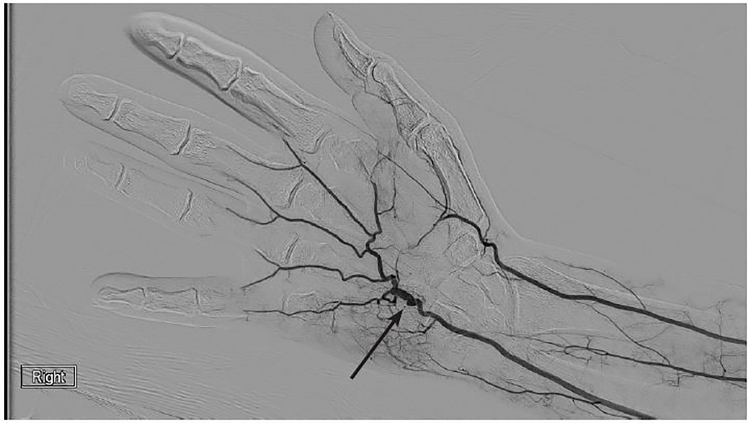
Angiogram demonstrating abnormal aneurysmal appearance of ulnar artery (arrow).

**Image 2b:**
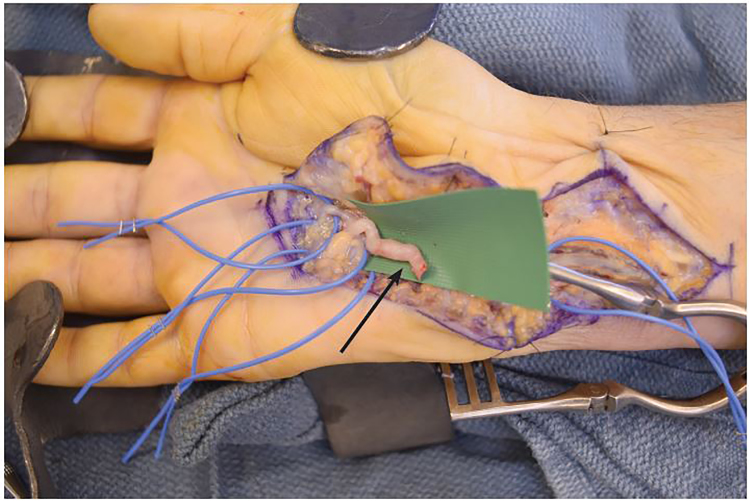
Intra-operative aneurysmal ulnar artery with corkscrew appearance (arrow).
